# Bacteriocins as Potential Anticancer Agents

**DOI:** 10.3389/fphar.2015.00272

**Published:** 2015-11-10

**Authors:** Sumanpreet Kaur, Sukhraj Kaur

**Affiliations:** Department of Microbiology, Guru Nanak Dev University, Punjab, India

**Keywords:** anticancer, cytotoxicity, bacteriocin, microcin, pediocin, cancer, nisin, colicin

## Abstract

Cancer remains one of the leading causes of deaths worldwide, despite advances in its treatment and detection. The conventional chemotherapeutic agents used for the treatment of cancer have non-specific toxicity toward normal body cells that cause various side effects. Secondly, cancer cells are known to develop chemotherapy resistance in due course of treatment. Thus, the demand for novel anti-cancer agents is increasing day by day. Some of the experimental studies have reported the therapeutic potential of bacteriocins against various types of cancer cell lines. Bacteriocins are ribosomally-synthesized cationic peptides secreted by almost all groups of bacteria. Some bacteriocins have shown selective cytotoxicity toward cancer cells as compared to normal cells. This makes them promising candidates for further investigation and clinical trials. In this review article, we present the overview of the various cancer cell-specific cytotoxic bacteriocins, their mode of action and efficacies.

## Introduction

Cancer is one of the major causes of morbidity and mortality throughout the world among the non-communicable diseases. In the year 2012, there were an estimated 14.1 million new cases of cancer and 8.2 million cancer deaths worldwide (Ferlay et al., 2013). Cancer cells are altered self cells which have escaped normal growth regulating mechanisms. Under normal conditions, balance is maintained between cell renewal and cell death and the production of new cells is regulated so that the number of a particular cell type remains constant. But due to environmentally-induced or inherited genetic mutations, cells stop responding to normal growth control mechanisms and give rise to clones of cells that expand to considerable size, producing tumor or neoplasm. Cancer cells show the following six essential alterations in cell physiology: self sufficiency in growth signals, insensitivity to growth-inhibitory signals, resistance to programmed cell death, limitless replicative potential, sustained angiogenesis and metastasis (Hanahan and Weinberg, 2011). The strategies available for the treatment of cancer are chemotherapy, surgery and radiation out of which chemotherapy is the main choice of treatment. But the conventional chemotherapeutic drugs which target actively dividing cells are often associated with drug-induced damage to healthy cells and tissues, as they do not specifically target the cancer cells. Secondly, cancer cells frequently become resistant to chemotherapy due to various factors such as increased expression of drug detoxifying enzymes and drug transporters, and due to increased ability to repair DNA defects in cellular machinery that mediate apoptosis (Raguz and Yagüe, 2008). Therefore, there is an urgent need for cancer cell-specific targeted therapies that can alone treat cancer or can be used as adjuvants to lower the therapeutic doses of conventional anticancer drugs. With the growing popularity of peptide therapeutics, the scientific community has started exploring bacteriocins as novel therapeutic agents against cancer. The toxic effects of bacteriocins on eukaryotic cells were observed for the first time by Farkas-Himsley and Cheung (1976). The effects of bacteriocins on mammalian cells have been reviewed earlier (Cornut et al., 2008). In this review, latest studies on the anticancer properties of bacteriocins have been summarized with particular emphasis on the studies that have shown selectivity of bacteriocins against cancer cells as compared to normal cells.

## Bacteriocins

Bacteriocins are ribosomally-synthesized cationic peptides that are produced by almost all groups of bacteria. The first bacteriocin was discovered in the year 1925 by Gratia from *Escherichia coli* (Gratia, 1925) and later named as colicin. Since then large number of bacteriocins have been identified from a diverse group of bacterial strains. Their physiological functions in bacteria seems to inhibit the growth of competing microorganisms in a particular biological niche by killing them (Nes and Holo, 2000). Most bacteriocins are extremely potent, exhibiting antimicrobial activity at nanomolar concentrations, as opposed to the peptide antimicrobials produced by eukaryotic cells, which normally have 10^2^–10^3^-fold lower activities (Jennsen et al., 2006). Interestingly, the producer cells are immune to their own bacteriocins (Cotter et al., 2005). The classification of bacteriocins has been revised from time to time. The latest classification arranges bacteriocins into three major classes based on their structural and physico-chemical properties (Zacharof and Lovitt, 2012).

## Classification of Bacteriocins

### Class I

Lantibiotics are small (<5 kDa) heat-stable peptides that are highly post-translationally-modified containing characteristic polycyclic thioether amino acids such as lanthionine, methyllanthionine, and the unsaturated amino acids such as dehydroalanine and 2-aminoisobutyric acid. Lantibiotics are further classified into two types depending on the difference in charge.

Type A lantibiotics such as nisin and lacticin 3147 are 2–4 kDa positively charged, screw-shaped, flexible molecules which causes pore formation in the cell membrane of the target organism and thereby leads to depolarization of the cytoplasmic membrane of the target species.

Type B lantibiotics are 2–3 kDa peptides having either no net charge or net negative charge. They are globular molecules, which interfere with cellular enzymatic reactions such as cell wall synthesis. Mersacidin secreted by *Bacillus* spp. is an example of this type (Sahl and Bierbaum, 1998).

### Class II

Class II bacteriocins are small (<10 kDa) heat stable, non-lanthionine containing peptides that are not post-translationally modified beyond the removal of a leader peptide and the formation of a conserved N-terminal disulfide bridge. They have amphiphilic helical structure, which allows them to insert into the membrane of the target cell, leading to depolarisation and death.

Subclass IIa bacteriocins such as, pediocin PA-1 and sakacin A, are monomeric and possess an N-terminal consensus sequence Tyr-Gly-Asn-Gly-Val-Xaa-Cys. They are active particularly against *Listeria monocytogenes*. Subclass IIb bacteriocins include lactacin F and lactococcin G. They are two-component bacteriocins, in which two separate peptides work synergistically to generate antimicrobial effect. Cotter et al. (2005) suggested the third subclass IIc that contains circular bacteriocins such as gassericin A, circularin A, and carnocyclin A. These peptides carry two transmembrane segments that facilitate pore formation in the target cells (Kawai et al., 2004). However, others have suggested considering circular bacteriocins as a separate class (Belkum et al., 2011).

### Class III

Class III bacteriocins are high molecular weight (>30 kDa) heat labile proteins. Some of the colicins, megacins (from *Bacillus megaterium*), klebicin (from *Klebsiella pneumonia*), helveticin I (from *L. helveticus*), and enterolysin (from *E. faecalis*) are the members of this group.

## Selectivity of Bacteriocins Toward Cancer Cells

Some of the bacteriocins (Table 1) as reviewed in the following sections have shown selective action toward cancer cells. Although the exact mechanism of the cancer cell specificity has not been studied but the various factors that could account for the selective action could be explained based on the generalized cell surface variations of cancer cells from the normal cells. The bilayered phospholipid membrane of normal mammalian cells is asymmetric with respect to the distribution of phospholipids on the inner and outer surface. The outer surface is made up of neutral choline-containing zwitterionic phospholipids such as sphingomyelin and phosphatidylcholine, whereas the inner surface has aminophospholipids such as phosphatidylserine and phosphatidylethanolamine. However, in cancer cells there is loss in asymmetry with respect to phospholipid types. Cancer cell membrane is known to carry a predominantly negative charge due to high levels of the anionic phosphatidylserine, O-glycosylated mucins, sialylated gangliosides, and heparin sulfates (Utsugi et al., 1991; Schweizer, 2009; Riedl et al., 2011). Bacteriocins are cationic peptides by nature and thus they preferentially bind to negatively-charged cell membrane of cancer cells as compared to normal cell membranes which are neutral in charge (Dobrzyńska et al., 2005; Hoskin and Ramamoorthy, 2008). Secondly, the selective binding of bacteriocins to cancer cells can be explained due to differences in the membrane fluidity of cancer cells. Cancer cells have higher membrane fluidity as compared to normal cells and this facilitates easy membrane destabilization (Sok et al., 1999). Lastly, the membranes of cancer cells contain a significantly higher number of microvilli compared to normal cells that increases the surface area of cancer cells (Chaudhary and Munshi, 1995) which results in the binding of more number of antimicrobial peptides to the cancer cell membrane as compared to normal cells (Chan et al., 1998a,b).

**TABLE 1:** **The bacteriocins having anticancer activities against various cancer cell lines**.

**Bacteriocin**	**Producer organism**	**Class**	**Size (kDa)**	**Cancer cell lines**	**References**
Colicin E3	*E*.* coli*	III	9.8	P388, HeLa, HS913T	Fuska et al. (1978); Smarda et al. (1978)
Colicin A	*E*.* coli*	III	>20	HS913T, SKUT-1, BT474, ZR75, SKBR3, MRC5	Chumchalova and Smarda (2003)
Colicin E1	*E*.* coli*	III	57	MCF7, HS913T	Chumchalova and Smarda (2003)
Microcin E492	*K*.* pneumoniae*	IIa	7.9	Hela, Jurkat, RJ2.25	Hetz et al. (2002)
Pediocin PA-1	*P*.* acidilactici* PAC1.0	IIa	3.5	A-549, DLD-1	Beaulieu (2004)
Pediocin K2a2-3	*P*.* acidilactici* K2a2-3	IIa	4.6	HT2a, HeLa	Villarante et al. (2011)
Pediocin CP2	*P*.* acidilactici*	IIa		HeLa, MCF7, Sp2/0-Ag 14, HepG2	Kumar et al. (2012)
Pyocin S2	*P*.* aeruginosa* 42A	III	74	HepG2, Im9 HeLa, AS-II, mKS-A TU-7	Abdi-Ali et al. (2004); Watanabe and Saito (1980)
Nisin	*L. lactis*	I	3.5	MCF7, HepG2	Paiva et al. (2011)
Bovicin HC5	*S*.* bovis* HC5	I	2.4	MCF7, HepG2	Paiva et al. (2011)
Smegmatocin	*M. smegmatis* 14468	III	75	HeLa AS-II, HGC-27 mKS-A TU-7	Saito et al. (1979); Saito and Watanabe (1979, 1981)
Plantaricin A	*L. plantarum* C11	II	2.4	Jurkat, GH_4_, Reh, Jurkat, PC12, N2A, GH_4_	Zhao et al. (2006); Sand et al. (2007); Sand et al. (2010); Sand et al. (2013)

## Colicins

Colicins are plasmid-encoded high molecular weight (>20 kDa) antimicrobial peptides secreted by *E. coli* and other related *Enterobacteriaceae*. They are active against *E. coli* strains and other closely related bacteria, such as *Salmonella* (Braun et al., 1994). Gratia in 1925 identified the first colicin, a heat labile product present in cultures of *E. coli* V. Gratia and Fredericq coined the term colicin in the year 1946, and demonstrated the proteinaceous nature and the activity spectra of colicins (Gratia and Fredericq, 1946). Thirty different types of colicins have been identified that are differentiated according to their killing activity and the mode of action (Smarda and Smajs, 1998; Lakey and Slatin, 2001). Colicin production by bacteria occurs principally during times of stress like nutrient or oxygen depletion and therefore are SOS regulated (Smarda and Smajs, 1998).

The mechanism of antimicrobial killing has been extensively studied in colicins and it has been shown that it kill target bacterial cell in three steps—cell binding, membrane translocation and cell death. The three different domains in the colicin perform these steps: The T (translocation) domain is N-terminally located, the R (receptor binding) domain is in the central region and the C (cytotoxic) domain is located at C-terminus. Colicins bind to the outer membrane proteins on target bacterial cells (James et al., 1996). Once bound to outer membrane, colicin enter cell by interacting with Tol or Ton complex of periplasmic proteins (Imajoh et al., 1982) and kill the sensitive target strain by one of the mechanisms- pore formation (colicins A, B, E1, Ia, Ib, K, L, N, U, 5, and 10), non-specific DNAse activity (colicins E2, E7, E8, and E9), or inhibition of protein biosynthesis by cleaving 16S rRNA or tRNAs (colicins E3, E4, E6, E5, and D) (Bowman et al., 1971; Cramer et al., 1990; Smarda and Smajs, 1998; Tomita et al., 2000; Lakey and Slatin, 2001).

Colicins are known to have anticancer activities against a variety of human tumor cell lines *in vitro* such as breast cancer, colon cancer, bone cancer and uteri cell line HeLa. Chumchalova and Smarda (2003) studied the inhibitory effects of four pure colicins—A, E1, U, and E3 on 11 human tumor cell lines with defined mutations of suppressor gene p53 and on one normal human fibroblast cell line MRC by MTT (tetrazolium bromide) assay. The effect of colicins on cell cycle was also studied. It was observed that colicin E1 and A had inhibitory effects on most of the cell lines. Colicin E1 showed 17–40% inhibition of all 11 cancerous cell lines with small differences among individual cell lines. The fibrosarcoma HS913T was most sensitive to colicin A, E1 and U showing 50% inhibition with colicin E1 treatment. The fibroblasts MRC5 was less sensitive to E1 than tumor cells. Colicin A had strong inhibitory effects varying from 16 to 56% inhibition of tumor cell lines but it also caused 36% inhibition of normal cell line MRC5. Breast carcinoma cell lines; BT474, ZR75, and SKBR3 were quite resistant to all tested bacteriocins and the inhibition ranged from 15 to 25%. Colicin E3 had no significant inhibitory effect against any cell lines tested.

Further, they studied colicins A-, E1-, and U–mediated cell cycle alterations in five selected cell lines (human breast cancer cell lines MCF7 and MDA-MB-231, Osteosarcoma cell line HOS, HS913T, and MRC5). Colicin A resulted in cell cycle alterations in both cancer cell line HST913T and normal cells MRC5; whereas, Colicin E1 treatment altered the cell cycle only in MCF7 line (number of cells in G1 phase got elevated by 26%). Colicins U and E3 did not show any significant change in cell cycle.

Furthermore, Colicin A resulted in an increase in proportion of cells undergoing apoptosis by 7–28% in all cell lines except HS913T. Apoptosis in MCF7 line was increased by 58% and in HS913T line by 14% after treatment with colicin E1. In a separate report, Colicin E3 exhibited 100% killing effect on human uteri carcinoma cell line, HeLa at the lethal units of 10^5^ (Smarda et al., 1978). Colicin E3 also showed time- and dose-dependent inhibitory effect on the proliferation of murine leukemia cells P388 (Fuska et al., 1978). The anticancer activity was observed only against the carcinoma cells at the doses that do not affect the normal cell line. In another study, three different murine lymphoma cell lines showed decrease in viability by 40–58% after treatment with colicin A and E2 (Smarda and Oravec, 1989). Again, the effect of colicins on tumor cells was highly specific. Colicins E1 and E3 were found to be cytotoxic for oncogene *v-myb-*transformed chicken monoblasts. However, colicin E3 did not cause any alterations in cell cycle which may indicate that it kill cells by necrosis rather than apoptosis (Smarda et al., 2001).

*In vivo* mice studies have also shown the protective anticancer effects of colicins. Direct injections of colicin E3 into the subcutaneous nodes of solid HK-adenocarcinoma showed 61% decrease in mean mass of tumor in mice (Cursino et al., 2002). Treatment with colicin A showed 43% prolonged survival of mice with transplanted LP-2 plasmacytoma (Chumchalova and Smarda, 2003).

The mechanism of cytotoxicity of colicins is not well understood. Few reports have shown that colicin E3 cleaves the 18S rRNA of the isolated eukaryotic ribosomes but whether rRNase domain of E3 is able to enter eukaryotic cells to allow it to attack ribosome is not known (Turnowsky et al., 1973; Suzuki, 1978). RNase and pore forming colicins kill tumor cells by generating pores in the plasma membrane. These pores activate apoptosis and many cells did not cross G1 phase of their cell cycle as shown in the previously referred study (Chumchalova and Smarda, 2003). It was also shown that colicin E3-treated cells had changes in their plasma membrane that lead to apoptosis (Chumchalova and Smarda, 2003).

## Microcins

Microcins are small sized (<10 kDa) bacteriocins secreted by enterobacteria (mostly *E. coli*) apart from colicins that are large sized proteins. They are secreted under conditions of nutrient depletion and exert potent antibacterial activity against closely related species. Fourteen microcins have been reported till date, out of which only seven have been isolated and characterized. The typical gene clusters encoding the microcin precursor, the self-immunity factor, post-translational modification enzymes and the secretion factor are located either on plasmids or on the chromosome. Class I microcins have the lowest molecular masses, ranging from 1 to 3 kDa, and display extensive post-translational modifications of their peptide backbone. Examples are B17, C7/C51J 25. The genes for all the three class I microcins are located on plasmids. Class II microcins are higher molecular mass microcins than class I microcins (4.9–8.9 kDa). Class II is further subdivided into two subclasses: class IIa, some of which contain disulfide bonds but no further post-translational modification (L, V, 24), and class IIb are linear microcins that may carry a C-terminal post-translational modification (E492, M and presumably H47 and I47).

Microcin E492 (M-E492) is low molecular mass (7887 Da) bacteriocin which is secreted by *Klebsiella pneumoniae* RYC492. It is known to have potent antimicrobial activity against number of pathogenic bacteria such as *E. coli*, *Klebsiella*, *Salmonella*, *Citrobacter*, and *Enterobacter* (Lorenzo, 1984). It acts by forming pores in the cell membrane of target cell and thereby disrupting the cell membrane potential (Lorenzo, 1984; Lorenzo and Pugsley, 1985; Lagos et al., 1993). M-E492 has modular structure and each module has specific function. The N- terminal module is responsible for its antibacterial activity and C- terminal is required for receptor recognition.

The toxic effect of M-E492 has been reported against various human malignant cell lines such as HeLa (human cervical adenocarcinoma), Jurkat (T cell derived from acute T cell leukemia), RJ2.25 (a variant of Burkitt’s lymphoma), and colorectal carcinoma cells. On the other hand, no effect was observed against normal bone marrow cells, splenocytes, KG-1, human tonsil cells and non-tumor macrophage derived cells (Hetz et al., 2002). The Jurkat was most sensitive of all the three cancerous cell lines with 96% loss in viability after 24 h of incubation with 14 μg/ml of M-E492. Further, it was shown that M-E492 caused apoptosis of cancerous cells at low concentrations (10 μg/ml) and necrosis at higher concentrations (20 μg/ml). Apoptosis is a preferred mode of cell death over necrosis as it is not known to induce inflammatory response. The prominent biochemical and morphological changes observed during apoptosis of cancer cells caused by M-E492 includes cell shrinkage, DNA fragmentation and extracellular exposure of phosphatidylserine. Also, apoptosis due to M-E492 was associated with the activation of caspases, loss of mitochondrial membrane potential, and release of calcium ions from intracellular stores (Hetz et al., 2002).

The induction of apoptosis of HeLa cells was 100 times better when the cells were incubated with M-E492 producer strain *E. coli* VSC257pJEM15 carrying the plasmid for MccE492 rather than the purified protein (Hetz et al., 2002). Thus, this shows an alternative way to deliver M-E492, provided the bacterial strain is non-toxic, non-immunogenic to the host, is able to replicate only in the tumor, and could be completely eliminated from the host. Interestingly, a study reported that the probiotic *E. coli* Nissle 1917 selectively colonizes tumors (and not healthy organs) when systemically administered to mice (Brader et al., 2008). *E. coli* Nissle 1917 has been used safely as probiotics under the name Mutaflor^®^, in humans for almost a century for the treatment of several intestinal disorders (Rembacken et al., 1999; Kruis, 2004). This strain produces both microcin M and microcin H47, however, the anticancer properties of these microcins are not known. Thus, it will interesting to test *E. coli* Nissle 1917 as delivery vehicle for the delivery of M-E492 in the tumor-bearing animal models.

The *in vivo* effects of M-E492 on human tumor cells were studied in a preclinical model in which human colorectal carcinoma xenografts were grown in nude mice. Preliminary results showed that M-E492 fibrils administered had antitumoral activity (Lagos et al., 2009). M-E492 is known to forms amyloid-like fibrils *in vitro*. Fibril formation is associated with the loss of antibacterial activity, however, it retains its ability to inhibit cancer cell lines. fibrils may be used as a depot for the sustained release of the peptides with biological activity (Turnowsky et al., 1973).

## Pyocins

Pyocins are produced by more than 90% of *Pseudomonas aeruginosa* strains and each strain may synthesize several pyocins (Michel-Briand and Baysse, 2002). A bacteriocin from *P. aeruginosa* 10 was isolated for the first time by Jacob (1954) after treatment of the cells with ultraviolet irradiation (mutagen). The pyocin genes are located on the *P. aeruginosa* chromosome and their activities are inducible by mutagenic agents such as UV radiations and mitomycin C (Kageyama and Egami, 1962). Three types of pyocins are known. (i) R-type pyocins resemble non-flexible and contractile tails of bacteriophages. All R-type pyocins are nuclease- and protease- resistant. They cause depolarisation of the cytoplasmic membrane with pore formation in target bacteria. (ii) F-type pyocins also resemble phage tails, but with a flexible and non-contractile rod-like structure. (iii) S-type pyocins are colicin-like, protease-sensitive bacteriocins. They are made of two components. The large component carries the killing activity (DNase activity for pyocins S1, S2, S3, AP41; tRNase for pyocin S4; channel-forming activity for pyocin S5).

Initially it was shown that partially purified pyocin obtained from *P. aeruginosa* under specific growth conditions, contain proteins that are lethal to L6OT mice fibroblast cell line (Farkas-Himsley and Cheung, 1976). Subsequently, Abdi-Ali et al. (2004) reported the cytotoxic effect of purified pyocin S2 and partially purified pyocin isolated from *P. aeruginosa* 42A on tumor cell lines –HepG2 (Human hepatocellular carcinoma) and Im9 (Human immunoglobulin-secreting cell line derived from multiple myeloma) and one normal cell line HFFF (Human fetal foreskin fibroblast). A dose-dependent inhibitory effect of Pyocin S2 was observed on tumor cell lines, whereas both pyocin S2 and partially purified protein was totally non-toxic to normal cell line HFFF. The cell line Im9 was more sensitive as compared to HepG2 and maximum growth inhibition of 80% was observed at maximum pyocin concentration of 50 U/ml after 5 days of cell incubation at 37°C and 5% carbon-dioxide.

The cytotoxicity of pyocin S2 was also observed by Watanabe and Saito (1980) against cancerous cell lines (HeLa, AS-II derived from embryonal carcinoma of ovary, simian virus-40 transformed mouse kidney cell line mKS-A TU-7) and normal mice cells (BALB/3T3). However, it had no cytotoxicity against some of the cancerous (HCG-27) and normal cells (rat kidney and human lung cells). The cytotoxicity was completely abolished by the addition of pyocin-sensitive cell membrane preparations, but not by adding cell membranes of pyocin-resistant cell. The periodate and neuraminidase treatment of pyocin-sensitive cell membrane preparations did not abolished the cytotoxicity of pyocin S2. Further, various sugars (D-galactose, *N*-acetyl D-galactosamine and *N*-acetyl neuraminic acid) neutralized the cytotoxicity-inhibitory activities of pyocin-sensitive cell membrane preparations, thereby hinting at the role of these sugar moieties on the cell membrane in their interaction with pyocin S2.

## Pediocin

Pediocins are small (>5 kDa), class IIa bacteriocins that are plasmid-encoded and produced by members of genera *Pediococcus* (Papagianni, 2003). They are thermostable peptides, active over a wide pH range, but sensitive to the action of many proteolytic enzymes like papain, pepsin, protease, trypsin and α-chymotrypsin (Kumar et al., 2011). Their N-terminal region contains the conserved Y-G-N-G-V/L “pediocin box” motif and two conserved cysteine residues that are joined by a disulfide bridge that forms a three-stranded antiparallel beta-sheet. The cationic N-terminal beta-sheet domain mediates binding of the class IIa bacteriocin to the target cell membrane; whereas the C-terminal region forms hairpin that penetrates into the hydrophobic region of the target cell membrane, thereby mediating leakage through the membrane (Fimland et al., 2005; Drider et al., 2006). There are various types of pediocins, such as pediocin L50, AcH, AcM, CP-2, F, K1, L, L-50, SJ-1, and many more. The types of pediocins, their characteristic properties, producing strains and their antimicrobial spectrum has already been reviewed (Papagianni and Anastasiadou, 2009; Kumar et al., 2011; Lohans and Vederas, 2012).

Pediocin PA-1 produced by *P. acidilactici* PAC1.0 and the recombinant pediocin cloned in *Pichia pastoris* inhibited the growth of cell lines A-549, a human lung carcinoma and DLD-1, human colon adenocarcinoma. Native pediocin PA-1 showed cytotoxic effect at very low concentrations of 1.6 μM whereas recombinant pediocin was ineffective at this concentration (Beaulieu, 2004). Another pediocin similar to PA-1 isolated from *P. acidilactici* K2a2-3, having molecular weight of 4.6 kDa was reported to have cytotoxic activities against HT29, a human colon adenocarcinoma and HeLa cell lines (Villarante et al., 2011). Using MTT assay, the study showed the inhibitory effect of dialyzed (800 AU/ml) and undialyzed (1600 AU/ml) bacteriocin fraction on the growth of cancer cell lines—HeLa and HT29. The percentage inhibition of HT29 observed with dialyzed and undialyzed fractions of bacteriocin were 53.7 ± 7.0, 55.0 ± 4.8, respectively. HeLa cell line was inhibited significantly more by undialyzed fraction (52.3 ± 6.0%) as compared to dialyzed fraction (15.6 ± 4.0%). In this study the specificity of the bacteriocin toward cancer cell lines was not tested. The mechanism of cytotoxicity was also not studied.

In yet another report, pediocin CP2 produced by *P. acidilactici* CP2 MTCC5101 and its recombinant version, synthetic fusion protein cloned in *E. coli* BL21(DE3)-*pedA*, were tested for their cytotoxic effects against various human cancer cell lines such as HepG2, HeLa and MCF7 (a mammary gland adenocarcinoma). The inhibition appeared to be dose-dependent as more inhibition was observed at 25 μg/ml than at 1 μg/ml. When treated with 25 μg/ml rec-pediocin and native pediocin CP2, MCF-7 cell lines retained the percentage viabilities of 2.13 and 10.74, respectively; whereas, HepG2 cell lines retained 5.52 and 1.23% viabilities, respectively (Kumar et al., 2012). HeLa cells appeared to be comparatively resistant to the effects of both rec-pediocin and native pediocin. The authors also reported that rec-pediocin caused apoptosis of the cancer cells after 48 h of incubation as studied by DNA fragmentation method.

## Nisin

Nisin is a low molecular weight pentacyclic antibacterial peptide of 34 amino acid residue produced by *Lactococcus lactis* subspecies *lactis* (Hansen and Liu, 1990). This bacteriocin belongs to the class lantibiotics. It consists of uncommon amino acids- lanthinone, methyllanthionine, didehydroalanine and didehydro-aminobutyric acid, introduced during post-translational modifications of protein (Klaenhammer et al., 1993). Nisin is a heat-stable bacteriocin (Jack et al., 1994) and has antimicrobial activity at nanomolar concentrations against wide range of Gram-positive bacteria and even against other lactic acid bacteria but no significant activity is reported against Gram-negative bacteria. Due to its non-toxicity in animals, World Health Organization, 1969 and Food and Drug administration (FDA) in 1988 (FDA, 1988) approved the consumption of nisin by humans as safe. It interacts with lipid II (Wiedemann et al., 2001), a membrane-bound precursor involved in cell-wall biosynthesis and generates pores in the target bacterial cells (Ruhr and Sahl, 1985; Sahl et al., 1987).

Paiva et al. (2012) demonstrated the cytotoxic effect of nisin to MCF-7 (human breast adenocarcinoma cell line) and HepG2. The IC_50_ (the concentration at which half of the cells are inhibited) values for both these cell lines were 105.46 and 112.25 μM, respectively. At the highest concentration tested, i.e., 140 μM, the cell viabilities of both the cell lines were found to be less than 20%. The reduction in cell viability of cancer cell lines was shown to be dose-dependent and at concentrations above IC_50_, cell shrinkage, vacuolization of cytoplasm, condensation and lateralization of nucleus was observed under the optical microscope ultimately leading to detachment of cells. The normal cell line was not used a controls to compare the cancer cell-specific activity. In yet another study (Begde et al., 2011), the IC_50_ cytotoxicity of nisin against Jurkat cell line was found to be 225 μM; however, the same concentrations were known to inhibit the normal human lymphocytes as well. However, no apoptosis of both the cancer cell line and normal cell lines were observed at the cytotoxic concentrations as investigated by DNA fragmentation assay (Begde et al., 2011). Maher and McClean (2006) reported the cytotoxicity of nisin against two human adenocarcinoma of colon and colorectum, HT29 and Caco-2 cell lines, and the IC_50_ values reported were 89.9 μM and 115 μM, respectively.

Joo et al. (2012) showed cytotoxicity of nisin in head and neck squamous cell carcinoma (HNSCC) both *in vitro* and *in vivo*. Three HNSCC cell lines UM-SCC-17B, UM-SCC-14A and HSC-3 were treated with nisin at different doses and primary human oral keratinocytes were used as control. Nisin at the doses ranging from 20 to 80 μg/ml showed significant increased apoptosis against all the three cancer cell lines as measured by DNA fragmentation and reduced cell proliferation as compared to the control oral keratinocytes. This cytotoxicity was conferred by apoptosis, cell cycle arrest and reduction in cell proliferation. Nisin was shown to cause apoptosis by alteration in calcium influx and cell cycle arrest in G2 phase as significantly higher calcium influx was observed in case of HNSCC cell lines as compared to primary keratinocytes. Further they employed Affymetrix gene array to study the effects of nisin on 39,000 genes. They identified that CHAC1, cation transport regulator and apoptosis mediator was fourfold upregulated in nisin-treated HNSCC cells. Furthermore, the *in vivo* effects of nisin was tested in oral xenograft mouse model. Nisin at the daily dose of 200 mg/kg for 3 weeks significantly reduced the tumor volumes as compared to controls.

In a very recent report (Kamarajan et al., 2015), the natural variants of nisin, nisin A and nisin Z were tested for cytotoxic effects on HNSCC cells both *in vitro* and *in vivo* in mice model. Both the variants of nisin showed significant dose-dependent decrease in the cell proliferation of the three cell lines (HSC-3, UM-SCC-17B, and UM-SCC-14A) at the doses ranging from 100 to 800 μg/ml, and similar dose-dependent effects were observed on the increase in number of apoptotic cells for two of the cell lines (HSC-3 and UM-SCC-17B). Apoptosis was determined by staining with annexin V and flow cytometry and by imaging cells with digital microscope after staining the cells with fluorescent dyes. Also nisin A and Z were tested for their efficacies in treating the tumors, established in the floor of mouth by subcutaneously injecting UM-SCC-17B cells. Oral gavage of nisin A and Z, at the dose of 800 mg/kg body weight per day for 3 weeks significantly reduced the mean mass of tumors by 3- and 17-fold respectively as compared to the control group gavaged with water only.

Preet et al. (2015) studied the synergistic effect of nisin along with doxorubicin on dimethylbenz (a) anthracene-induced skin carcinogenesis in mice. They showed that nisin and doxorubicin alone reduced the mean tumor volumes by 14 and 51.3%, respectively after 4 weeks of treatment. Whereas the combination of nisin-doxorubicin reduced the tumor volume by 66.82% as compared to the untreated group. In nisin-doxorubicin treated group, cells showed chromatin condensation and marginalization of nuclear material which might be due to apoptosis in tumor tissues. From this study they concluded that nisin has ability to increase the potential of chemotherapeutic drug, doxorubicin.

## Plantaricin A

Plantaricin A (plnA), produced by *Lactobacillus plantarum* C11 is a 2.4 kDa class II antimicrobial bacteriocin having pheromone-like activity. It exists in three variable forms: a 26 residue peptide and two N-terminally truncated forms containing 23 and 22 residues. All the three variants are derived from 48-residue precursor encoded by *pln*A gene (Diep et al., 1996). It possesses antimicrobial activity against closely related species of lactic acid bacteria in the pH range 4.0–6.5 (Daeschel et al., 1990). The amphiphilic nature of plnA results in its oligomerization into the cell membrane of the target bacteria and thereby forming pores (Nissen-Meyer et al., 1993).

Zhao et al. (2006) reported the dose- and temperature-dependent cytotoxicity of artificially synthesized plnA against human T cell leukemia cell line, Jurkat, *in vitro*. The cell viability decreased by 75% after treatment with 25 μM plnA at 20°C; whereas at 37°C the cell viability was reduced by 55%. The mode of killing appeared to be apoptosis along with necrosis as observed microscopically by fragmentation of cell nuclei and plasma membrane of cancer cells. The intracellular concentration of caspase-3 in jurkat cell line was also increased upon treatment with plnA. Further the effect of lipid composition of liposomes on the interaction of plnA with liposomal membranes was studied (Zhao et al., 2006). PlnA readily permeabilized the liposomes containing negatively charged phospholipids as shown by leakage of fluorescent dye carboxyfluorescein, and secondly it was shown that plnA forms amyloid like fibrils upon binding with negatively charged phospholipids, such as phosphatidylserine (Zhao et al., 2004, 2006). Thus, the negatively charged lipids of target cell membrane might promote the association of plnA and concentrate it on the target cell membrane. The study concluded that the concentrating effect of the phospholipids was responsible for effectiveness of these peptides at nanomolar concentrations and fibril formation could be important for its cytotoxicity. A significant increase in the exposure of phosphatidylserine on the surface of various types of cancer cells have been reported earlier (Riedl et al., 2011), that might be responsible for enhanced interaction of plnA with tumor cells.

In another report (Sand et al., 2007), the effect of artificially-synthesized plnA on the permeabilization of normal as well as cancerous rat anterior pituitary cells (GH_4_ cell line) was studied by using whole-cell patch-clamp recordings of the membrane potential and also by microfluorimetry that measured cytosolic Ca^2+^ concentration by using Ca^2+^-sensitive fluorochrome fura-2. PlnA in a dose-dependent manner differentially permeabilized GH_4_ cell line whereas normal cells were resistant. GH_4_ cells were exposed to different concentrations of plnA by using pressure ejection. At 10 μM plnA concentration, 12 cells out of 25 got permeabilized; at 100 μM, 27 out of 29 were permeabilized within few seconds of exposure and at 1 mM concentration all cells were instantly permeabilized; whereas, plnA had no permeabilizing effect on normal cells even at the concentration of 1 mM. However, in a separate study, Sand et al. (2010) did not find any significant differential effect of plnA on normal and cancerous cell lines. The cytotoxicity of plnA was studied on normal human B and T cells, Reh cells (human B cell leukemia cell line), Jurkat cells, normal rat cortical neurons and glial cells, PC12 (rat adrenal chromaffin tumor), and murine N2A cell lines (spinal cord tumor cell line). PlnA at 20 μM concentration caused 100% cell death of cancerous cell lines, Reh and Jurkat; whereas 100% cell death of normal cell lines occurred at 40 μM concentration. The difference in the dose for the cell death of cancer and normal cells was non-significant for selectively targeting cancer cells under physiological conditions. Thus, structural modifications of plnA should be tested for their selectivity to cancer cells. But, rational modification warrants the knowledge of the molecular mechanism of cytotoxicity of plnA. Thus, to identify the surface molecules on the eukaryotic cells critical for the interaction of plnA with eukaryotic cell, further studies were done (Sand et al., 2013). The group reported that the membrane permeabilizing effect of plnA in case of eukaryotic cell membranes is dependent on the negative surface charge conferred by membrane glycosylated proteins. The permeabilizing effect of plnA was observed at the concentration of 100 μM in GH_4_ cells by using patch clamp membrane conductance recordings and microfluorometric techniques. However, the permeabilizing effect of plnA was drastically reduced on neutralizing the negative surface charge of cells by the addition of Ca^2+^ or poly-D-lysine to GH_4_ cells. Further, to test the role of surface glycoproteins in cell membrane permeabilization, GH_4_ cell patches were treated with trypsin for 1 min and then studied the membrane permeabilization. Trypsin-treated GH4 cells were resistant to plnA at 100 μM concentration. Similarly, removal of carbohydrate residues from glycosylated membrane proteins by exposing the cells to a mixture of enzymes—PNGase F, chondroitinase ABC and heparinase I, II, III made the cells resistant to plnA-induced permeabilization. Thus, this study shows the role of glycosylated membrane proteins in plnA-induced membrane permeabilization. The contrasting results were obtained in another study, wherein the effects of bacteriocins pln C, nisin A and pediocin-1/AcH were tested for their cytotoxicities against cancer cell lines HeLa and HT29 (Martín et al., 2015). All the three bacteriocins tested at concentrations 1 and 10 μg had no cytotoxic effects against the cell lines HT29 and HeLa; however, enzymatic treatment (chondroitinase ABC and heparinase I, II, III) of the cell lines prior to bacteriocin addition resulted in loss in cell viabilities by almost 70% as shown by trypan blue assay. Thus, the study concluded that glycosaminoglycans on the eukaryotic cell surface are negatively charged that binds to the positively charged bacteriocins and neutralize them. However, removal of the glycosaminoglycans resulted in making the cell lines susceptible to the cytotoxic effect of the bacteriocins. PlnC is a 3.5 kDa lantibiotic produced by *L. plantarum* LL441 (Turner et al., 1999). The study used very low concentration of plnC, i.e., 10 μg that amounts to 2.86 × 10^–6^ μM as compared to the previous studies in which 100 μM plnA was used. The high concentrations of the bacteriocins may have entirely altered cytotoxic effects on the cell lines. Also cell-specific cytotoxic effects of bacteriocins is already known. Thus, difference in the bacteriocin concentration and eukaryotic cell types used in both the experiments may to some extent explain the contrasting results obtained. Nevertheless the role of glycosaminoglycans in interacting with bacteriocins remains unresolved.

## Bovicin

Bovicin is a lantibiotic produced by *Streptococcus bovis* HC5 having molecular weight of 2.4 kDa. It is stable to autoclaving and low pH and is structurally and functionally similar to nisin. It has broad-spectrum antimicrobial activity against closely related *S. bovis* strains as well as various other Gram-positive and Gram-negative bacteria but do not inhibit *E. coli* K-12. The activity is resistant to proteinase K and α-chymotrypsin but sensitive to pronase E and trypsin. The antimicrobial activity is due to pore formation in cell membrane and inducing potassium efflux in the target cells (Mantovani et al., 2002). Paiva et al. (2012) reported the cytotoxic activity of bovicin HC5 *in vitro* against human cell lines-MCF7 and HepG2. The IC_50_ values of bovicin for both these cell lines were 279.39 μM and 289.3 μM, respectively. At the highest concentration tested, i.e., 350 μM, the cell viabilities of both the cell lines were found to be less than 20%.

## Smegmatocin

Smegmatocin 14468 is a 75 kDa bacteriocin produced by *Mycobacterium smegmatis* 14468 and is known to have narrow spectrum activity against *M. diemhoferi* ATCC 19340 (Saito et al., 1979). Smegmatocin is not secreted by the bacteria in the culture supernatant. Therefore to purify smegmatocin, the cells are ultrasonicated to release the bacteriocin in the culture supernatant. The smegmatocin is heat labile and inactivated at 100°C; 10 min treatment. It is also inactivated by proteolytic enzymes such as pepsin, trypsin and chymotrypsin (Saito et al., 1979). Further, Saito et al. (1979) also showed that smegmatocin inhibited the human cell line HeLa S3. Treatment of HeLa S3 cells with smegmatocin at a concentration of 64 and 128 AU/ml for 96 h caused almost 1 log decrease in the number of viable cells as compared to control heat-inactivated smegmatocin preparation. The cancer cells showed morphological changes, such as shrinking and appearance of vacuoles in their cytoplasm (Saito et al., 1979).

In another report, treatment of mKS-A TU-7 cells with as low as 32 AU/ml of smegmatocin for 12 to 24 h led to significant decrease in the number of cells as compared to normal untransformed cells. The reduction in the number of cells was associated with the reduction of both protein and DNA synthesis. The synthesis of DNA in the transformed cells almost ceased after 6 h of smegmatocin exposure; whereas in the controls the synthesis continued (Saito and Watanabe, 1981). In yet another report, AS-Il cells and HGC-27 cells from metastatic lymph node of gastric cancer were treated with smegmatocin 14468. The smegmatocin showed dose-dependent lethal effects on the cancer cell lines. The cells of AS-II were more sensitive to smegmatocin as compared to HGC-27 cell line (Saito and Watanabe, 1979). Further investigations are required to determine the mechanism of action of smegmatocin against human cancer cell lines because smegmatocin is quite a large protein and its activity and uptake inside the eukaryotic cell is not known. Interestingly, live *M. smegmatis* has shown immunomodulatory protective effects against mice model of cancer (Young et al., 2004; Rich et al., 2012), however, the role of smegmatocin in the cancer mouse model was not studied. Hence it will be interesting to compare the protective effects of smegmatocin-producing and non-producing *M. smegmatis* bacterial cells on the cancer cells, *in vivo*.

## Conclusion and Future Perspectives

Some bacteriocins have shown *in vitro* cytotoxicity against cancer cells at nano or micromolar concentrations. The cytotoxicity of the bacteriocin and its ability to differentially target cancer cells may depend on its structural properties such as number of positively charged amino acids, hydrophobicity and ability to form amphipathic structures or oligomerization as in case of other antimicrobial peptides (Gaspar et al., 2013). The cell membrane appears to be the major target of bacteriocins in eukaryotic cells. The enhanced expression of negatively charged cell surface molecules on the cancer cells makes them prone to the cytotoxic activity of the bacteriocins (Zhao et al., 2006). The mechanisms of cytotoxicity of bacteriocins include induction of apoptosis and/or depolarisation of the cell membrane leading to permeability changes. Few of them may induce both necrosis and apoptosis. The membrane potential measurement studies have shown that within few seconds of the interaction of bacteriocin with the sensitive eukaryotic cell, depolarisation of its surface and increase in its permeability occured leading to the cell death. The rapid killing induced by cytotoxic bacteriocins might indicate the non-receptor-mediated mode of action. The membrane surface glycoproteins and glycosaminoglycans were shown to be the cancer cell targets at least in case of plnA (Sand et al., 2013); however, in case of other bacteriocins, such as nisin A, plnC and pediocin glycosaminoglycans appeared to prevent the cytotoxic action on the cancer cells (Martín et al., 2015). Further studies are required to substantiate the role of surface molecules in their interaction with other cytotoxic bacteriocins. The anticancer potential of bacteriocins has been tested under laboratory conditions. However no data is available regarding their efficacies in cancer patients. The advantages of bacteriocins as therapeutic agents are that they are small peptides and thus are mostly non-immunogenic by nature (Bhunia et al., 1990). Secondly they are biodegradable and easily hydrolysed to simple amino acids, although this also means that they may not be as long-lasting. Due to these safety aspects, nisin has been approved for use as food preservative in more than 50 countries worldwide and it holds a GRAS (generally regarded as safe) status by WHO (Deegan et al., 2006). One of the major challenge for the use of bacteriocins as drugs is to improve the stability of bacteriocins in human gut or tissues. Efforts have been made to chemically synthesize bacteriocin peptides incorporating D-amino acids that are less susceptible to proteolytic cleavage in the gut. Analogs of lactococcin G were synthesized with the N- and C-terminal residues replaced with D-amino acids that were less susceptible to exopeptidases without much effect on their activities (Oppegård et al., 2010). In another study, site-directed alteration of trypsin recognition sites in salivaricin P resulted in trypsin-resistant variants with only slight variation in its anti-listerial activity (Shea et al., 2010). Similar studies aimed at enhancing the stability and potency of anticancer bacteriocins is warranted. Further, the functional vehicles for the targeted and controlled delivery of bacteriocins could also improve their *in vivo* stabilities.

Another hurdle in the commercialisation of therapeutic bacteriocins is the production and purification of bacteriocins on a large scale. The bacteriocins are purified from the culture supernatant by using a combination of cationic and hydrophobic chromatography and the typical concentrations of purified bacteriocins are less than a milligram per liter of the culture (Carolissen-Mackay et al., 1997). However, the knowledge about the genetic organization and biosynthetic pathways of various bacteriocins has facilitated the heterologous production of bacteriocins in different hosts as fusion proteins. It has simplified the purification protocols and enhanced the bacteriocin production. The details of heterologous production of bacteriocins are reviewed earlier (Rodríguez et al., 2003; Lohans and Vederas, 2012). Further, as bacteriocins are non-immunogenic, biodegradable and shown to have the cancer cell-specific toxicities, their potential to serve as synergistic agents to conventional cancer drugs should also be tested. The stage is now set where the potential of bacteriocins as anticancer agents should be harnessed for designing safer and better therapies for the mankind.

### Conflict of Interest Statement

The authors declare that the research was conducted in the absence of any commercial or financial relationships that could be construed as a potential conflict of interest.
